# Supplementary material for: Curbing household food waste and associated climate change impacts in an ageing society
Source: Nat Commun. 2024 Oct 21;15:8806. doi: 10.1038/s41467-024-51553-w (PMC11494014; doi:10.1038/s41467-024-51553-w)
Supplement: Supplementary file 1 — Supplementary Information [file 41467_2024_51553_MOESM1_ESM.pdf]

Supplementary Information:

Curbing household food waste and associated climate change impacts in an ageing society

Yosuke Shigetomi [a*], Asuka Ishigami [b], Yin Long [c], Andrew Chapman [d]

a) Faculty of Science and Engineering, Ritsumeikan University, 1-1-1 Nojihigashi, Kusatsu, Shiga, 525-8577, Japan
b) Faculty of Environmental Science, Nagasaki University, 1-14 Bunkyo-machi, Nagasaki, 852-8521, Japan
c) Graduate School of Engineering, The University of Tokyo, 7-3-1 Hongo, Bunkyo-ku, Tokyo 113-8654, Japan
d) International Institute for Carbon Neutral Energy Research Kyushu University, 744 Motooka, Nishi-Ku, 819-0395, Japan

*Corresponding author: y-shig@fc.ritsumei.ac.jp

<Keywords>

aging population, household food waste, greenhouse gas emission, life cycle assessment, Japan

Supplementary Figures

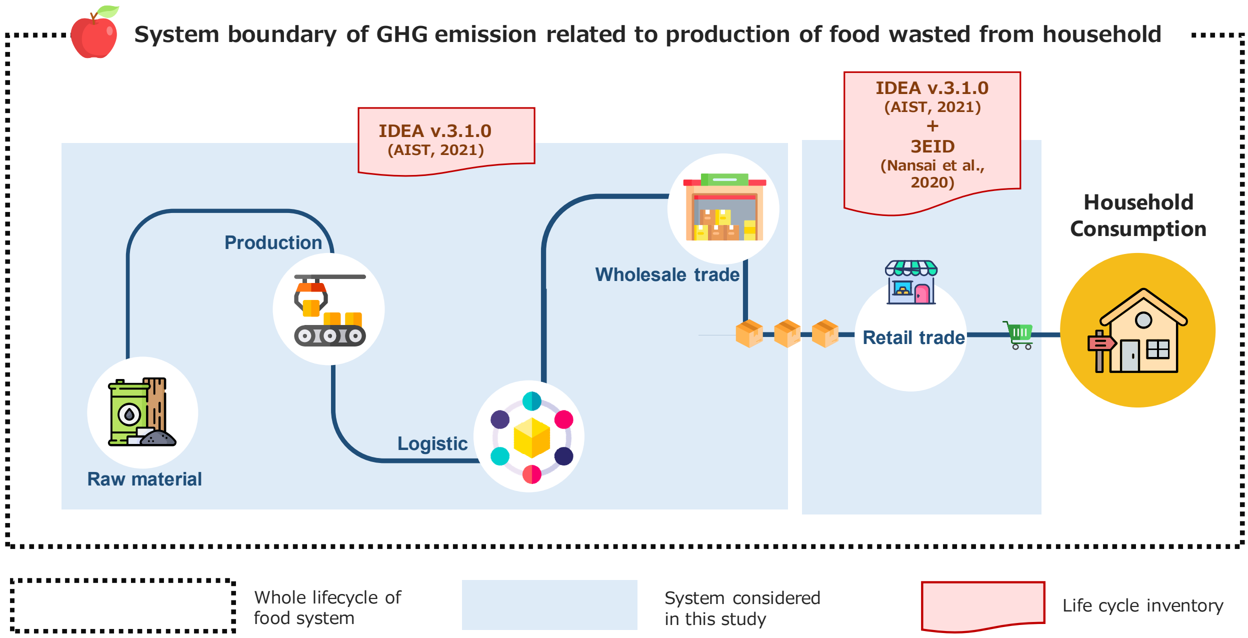

Supplementary Fig. 1. Food life cycle and the system boundary of the FWGHG in this study.

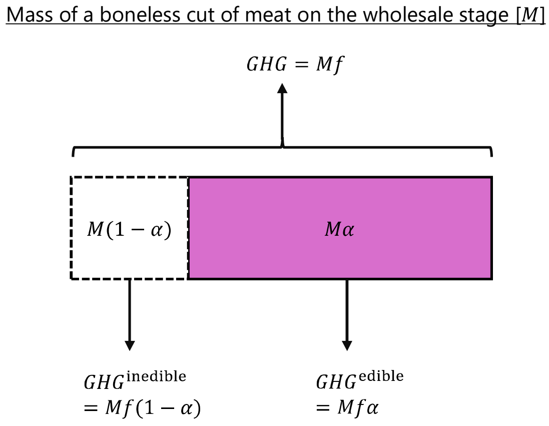

Supplementary Fig. 2. A schematic figure of the calculation of life cycle GHG emissions for food on the wholesale stage. GHG, GHG^{edmible}, and GHG^{inedible} represent the life cycle GHG emissions based on the mass of food. f represents the life cycle GHG emission intensity at the wholesale stage. M and α ($0 < \alpha \leq 1$) represent the mass of food and the yield ratio of the edible part at the wholesale stage, respectively.

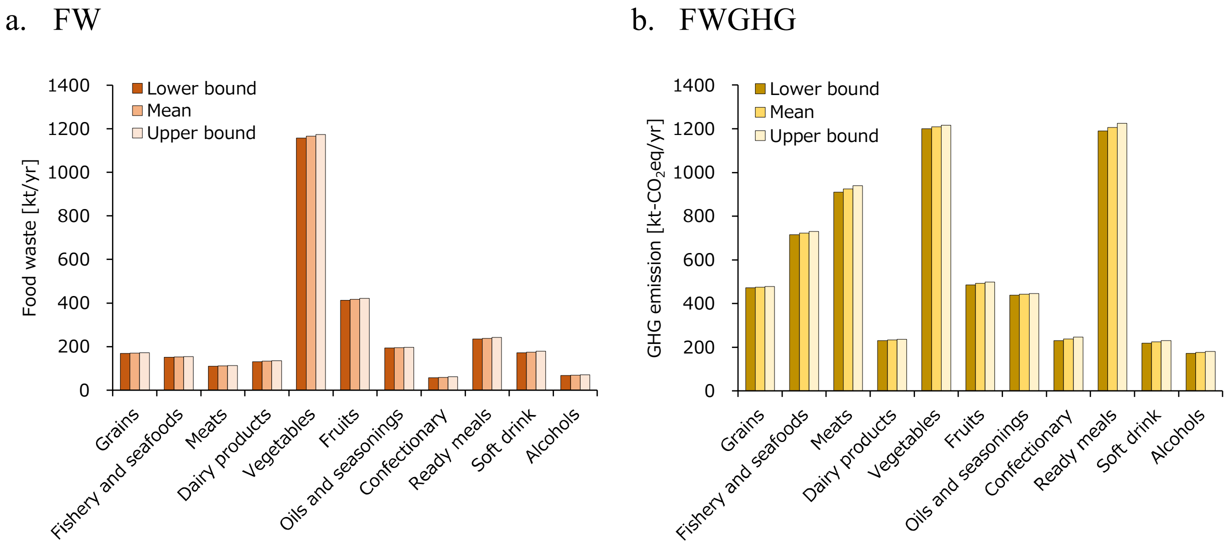

Supplementary Fig. 3. Comparisons of household FW (a) and FWGHG (b) for the three different waste ratios across food categories. “Mean” denotes the result of the mean FW ratios across the three age brackets. “Upper bound” and “Lower bound” denote the cases in which the FW ratios were taken between the maximum and minimum values across the three age brackets on FLSS,
29 respectively.

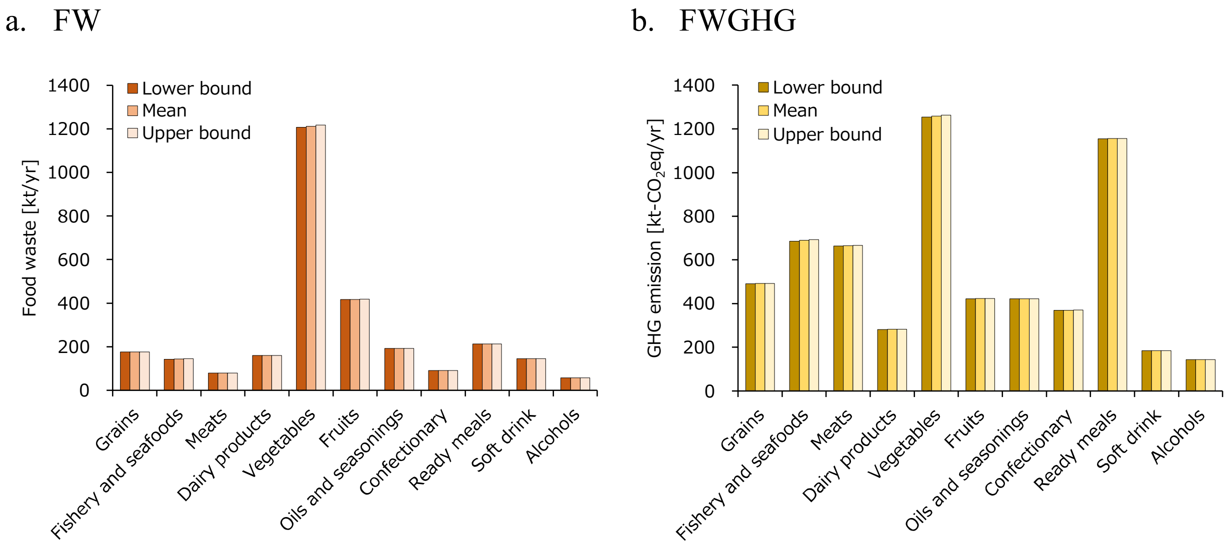

Supplementary Fig. 4. Comparisons of household FW (a) and FWGHG (b) for the three different inedible ratios across food categories. “Mean” denotes the result of the mean inedible ratios. “Upper bound” and “Lower bound” denote the cases in which the inedible ratios were taken between the maximum and minimum values by food commodity on the STFC, respectively.

30

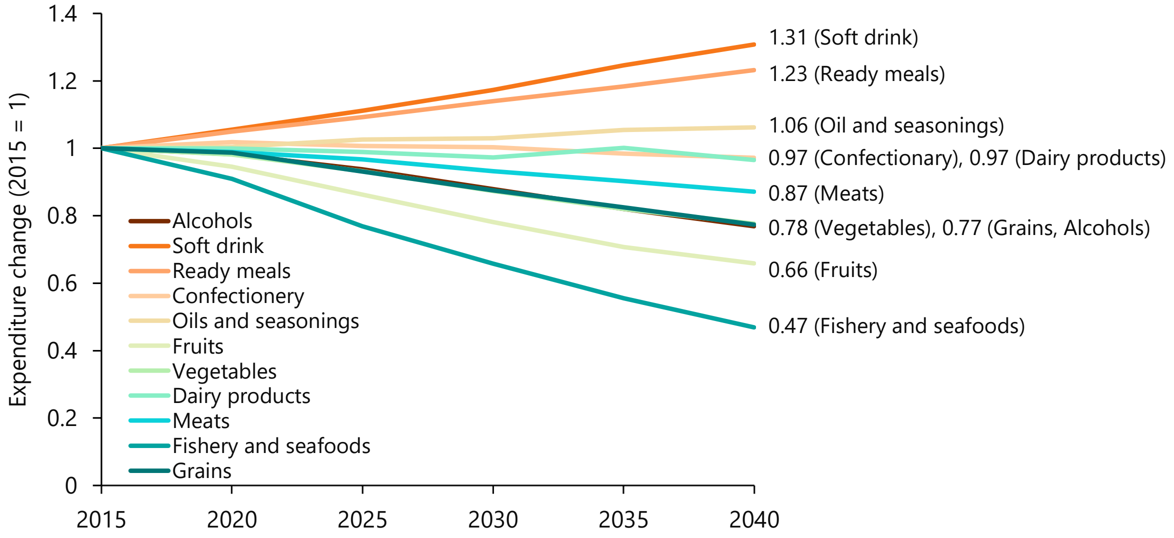

Supplementary Fig. 5. Future trends in the food expenditure changes being estimated by PRIMAFF (2019) (2015=1)

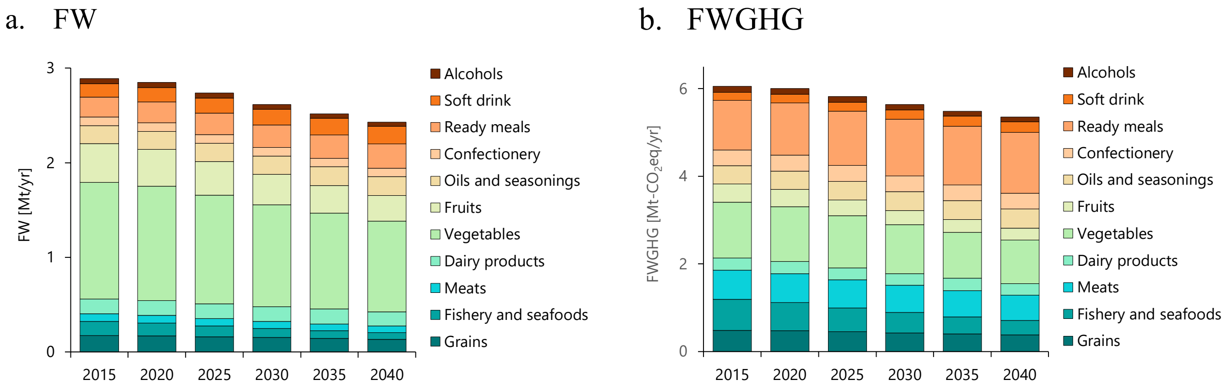

Supplementary Fig. 6. Future projections of household FW and FWGHG by using the food expenditure changes being estimated by PRIMAFF (2019) and these intensities calculated in this study.

Supplementary Table 1. Numbers of the mean family sizes from 2015 to 2040 estimated in this study

Year	20s	30s	40s	50s	60s	70s
2015	1.55	3.10	3.29	2.72	2.18	1.64
2016	1.55	3.09	3.27	2.71	2.17	1.62
2017	1.55	3.09	3.27	2.70	2.17	1.60
2018	1.55	3.09	3.26	2.69	2.17	1.58
2019	1.55	3.09	3.26	2.69	2.17	1.57
2020	1.55	3.09	3.26	2.68	2.17	1.56
2021	1.55	3.09	3.26	2.68	2.17	1.55
2022	1.55	3.09	3.26	2.67	2.17	1.54
2023	1.55	3.09	3.26	2.66	2.17	1.53
2024	1.54	3.09	3.26	2.65	2.17	1.52
2025	1.54	3.09	3.26	2.64	2.17	1.51
2030	1.54	3.09	3.26	2.62	2.16	1.47
2035	1.54	3.09	3.26	2.62	2.13	1.47
2040	1.54	3.09	3.26	2.62	2.13	1.46

Supplementary Methods

Method to ensure the system boundary of FWGHG from raw material to retail trade

It should be noted that the inventory data of IDEA [2] for agricultural and fishery products are based on the weight at the wholesale stage. However, the weight and physical units used in this study are based on the retail stage. This may lead to an underestimation of FWGHG. Hence, we combined the information based on an environmental input-output analysis (EIOA) to fill the emissions gap between wholesale to retail stages of agricultural and fishery food waste.

The Embodied Energy and Emission Intensity Data for Japan Using Input-Output Tables (3EID) [3] provides the direct GHG emission coefficients for commodities based on Japan's input-output table (JIOT). Combining JIOT and 3EID allows us to calculate the breakdown of emissions from the production to the retailer (i.e., emissions related to the margins). Since it is extremely difficult to transform the food weights at the retail stage that were estimated in this study to those at

the wholesale stage, we calculated the emission ratio of the total emissions from production to retail stages to those from production to wholesale stages and multiplied it by the corresponding emission intensity (intensities) in IDEA. This procedure aims to complement the emission intensity data in IDEA from wholesale to retail stages.

Method to determine the survival ratios of food

FLSS [4] indicates the intake and waste of *edible* food while our estimation of the amount of food consumption by household attribute focuses on a portion of a whole food (*edible* + *inedible*). Hence, to fill these weight gaps, we referred to STFC [5]. By combining this ratio with the weight of food purchased by the households which was quantified in the manuscript, we have estimated the weight of the edible part. The methodology is as follows.

First, we manually matched our food commodities based on the FIES [6] with the food items from the STFC in as detailed a manner as possible. When the food commodity i from FIES can be attributed to food item(s) l, that is $i \in l$, the refuse rate on commodity i, ρ_i was calculated as:

$$\rho_i = \frac{\sum_l \rho_l}{n_i} \tag{S1}$$

where n_i denotes the number of attributable commodities. Hence, the above equation represents the arithmetic mean value of the refuse rates. We recognize that this assumption affects the determination of ρ_i, however, there is currently no information about consumption amounts of each food item according to STFC, to our best knowledge. Therefore, we also prepared $\rho_{i,\max}$ and $\rho_{i,\min}$ by retrieving the highest and lowest refuse rates among food items related to food commodity i.

$$\rho_{i,\max} = \max_{i \in l} \rho_l \tag{S2}$$

$$\rho_{i,\min} = \min_{i \in l} \rho_l \tag{S3}$$

For example, *sea bream* (*tai*, in Japanese) on FIES can be attributed to the four different species and two different types (natural or aquaculture) including fresh processing (i.e., sashimi) (therefore, six items on STFC are attributed to *sea bream* on FIES), ranging from 0 to 60% refuse ratios. Then, ρ_i, $\rho_{i,\max}$, and $\rho_{i,\min}$ represent 45.8, 60, and 0. ρ_i was utilized for presenting the results in the main text and both $\rho_{i,\max}$ and $\rho_{i,\min}$ for quantifying their uncertainty in terms of the inedible ratio (for details, see the next section).

Moreover, some commodities; *beef*, *pork*, and *other raw meats* need the additional consideration because their emission intensities focus on the life cycle GHG of the boneless cut of

meat at the wholesale stage. While the boneless cut of meat is processed to the dressed meat for the retailer, the edible and inedible parts are engendered. Our objective is to quantify the responsible GHG emissions of households related to their FW. Therefore, we adjusted the emission intensities of *beef*, *pork*, and *other raw meats* by the following method.

The MAFF reports a rough estimation of yield ratios for beef (Japanese beef: wagyu beef) and pork from these boneless cuts of meats to dressed meats, accounting for 0.909 (= 300 / 330 kg) and 0.833 (= 50 / 60 kg) in the latest year implicitly, respectively [7]. Thus, we adopted these values in our analysis and recalculated the FWGHG. The mass balance and the life cycle GHG emissions on the wholesale stage are illustrated in Supplementary Fig. 2. When the life cycle GHG emissions of food on the retail stage should be attributed to its edible part, the adjusted emission intensity can be described as $f^{\mathrm{adj}} = \frac{GHG}{M\alpha} = \frac{f}{\alpha}$. Now, $\alpha_{\mathrm{beef}} = 0.909$ and $\alpha_{\mathrm{pork}} = 0.833$. For *other raw meats* than *beef* and *pork*, the yield ratio $\alpha_{\mathrm{other\ raw\ meat}}$ would be much lower than α_{beef} and α_{pork}. However, we could not find a good reference for this ratio and determined it the arithmetic sum of the yield ratios of beef and pork as the same method of calculating the emission intensity in IDEA. In addition, the yield ratios of imported raw meats are assumed to be same because it is not possible to distinguish the domestic and imported food as already mentioned in the limitation. Thus, addressing these assumptions will be a future direction.

Supplementary Discussion

Sensitivity analyses of FW and FWGHG with respect to food waste and inedible ratios

As outlined in the main text, representing the results of any uncertainty or sensitivity is essential. If the distributions of per-household food consumption, waste patterns, prices of food commodities, and the ranges of the life cycle inventory of food can be obtained, it is possible to conduct a detailed sensitivity analysis. However, these (micro) data are unavailable. Therefore, we considered the FW ratio and the inedible ratio for the uncertainties faced in this study and conducted the Monte Carlo simulations (N=1,000) to estimate the variations and 95% confidence intervals (95%CI) of FW and FWGHG with respect to each of them, respectively. Nevertheless, the discussion and conclusion that are detailed in the manuscript are not seriously affected even after considering the below changes.

(a) FW ratio based on FLSS

FLSS also presents the household FW compositions per food consumed by single (36), two-person

(168), and more than three-person households (142), respectively (the numbers in parentheses denote the number of sample households). Those food waste compositions recorded in FLSS imply that the FW ratios per category among the household brackets do not necessarily correlate with the family size. Hence, we assumed the possible FW ratios for each food category would vary between the lowest and highest ratios that can be calculated by summations of the maximum and minimum ratios of FW by kind of waste, respectively.

The result presents the total FW and FWGHG accounted for 2.89 ± 0.032 Mt/yr and 6.34 ± 0.084 Mt-CO_2eq/yr, respectively. The reason why the total FWGHG were larger than that of the main result (6.06 Mt-CO_2eq/yr) is that the FW composition was changed from the main result (i.e., when using the mean FW ratios across all of the sample households on the FLSS). For the food commodities, Most of them showed 0~2% changes in their FW and FWGHG, and some related to ready meals represented around 3%. However, these changes are trivial enough to keep the discussion regarding the importance of food for FW and FWGHG that we have already described in the main text. Supplementary Fig. 3 represents the ranges of predicted FW and FWGHG for 11 food categories, showing the sensitivities. The detailed results (relative) are shown in Supplementary Data 2.

(b) Inedible ratio based on STFC

Supplementary Fig. 4 summarizes the calculation results of FW and FWGHG for 11 food categories with respect to these uncertainties associated with ρ_i. Then, the total FW and FWGHG accounted for 2.89 ± 0.003 Mt/yr and 6.09 ± 0.014 Mt-CO_2eq/yr, respectively. Compared to the sensitivity result of the FW ratio described above, the ranges of FWGHG were smaller. The detailed results (relative) are shown in Supplementary Data 2.

Projections of FW and FWGHG based on the future trend of food consumption estimated by the governmental report

As mentioned in the Discussion section, food consumption is affected by a change in various factors including household income, food price, dietary trends over time, cohort, and so on. Again, it is currently difficult to clarify the sensitivities associated with these factors. The Policy Research Institute of the Japanese Ministry of Agriculture, Forestry and Fisheries (PRIMAFF) publishes a report that estimates the future trend in food consumption expenditures [1]. The report presents the estimation of changes in food consumption expenditures (monetary based) for three groups; fresh

foods, processed foods, and restaurants from 2015 to 2040 (2015=100). In addition, it shows the compositions of food consumption expenditure for 11 categories every five years from 2015 to 2040, which are the same categories and analytical period adopted in this study. With these two pieces of information, we obtained increase/decrease ratios of the food consumption expenditures for the categories based on the 2015 values as shown in Supplementary Figure 5.

Further, we attempted to project the FW and FWGHG from 2020 to 2040 by combining these ratios and the FW and FWGHG intensities by category obtained in this study if the food consumption amounts increase in line with the expenditures (therefore, the food prices are assumed to be constant from 2015) as is implied in the reference. Overall, the FW and FWGHG were expected to decrease markedly compared to the initial projections in this study (see Supplementary Figure 6). In 2040, FW and FWGHG were estimated at 2.43 Mt/year (-16.0% compared to 2015) and 5.35 Mt-CO_2eq/year (-11.7%), respectively. These reasons are explained mainly by expectations that dependencies on processed foods and restaurants would grow in contrast to fresh foods due to increases in single and elderly households who are more likely to prefer these foods [1].

However, it is extremely difficult to reproduce the methodology to estimate future trends in food consumption expenditure since the detailed results of trends in food consumption expenditure and the material and methods are not sufficiently presented in the reference. Therefore, we showed this finding as a complementary evidence base and sensitivity measure for this study in the Supplementary Information.

Supplementary Reference

1. Policy Research Institute of the Ministry of Agriculture Forestry and Fisheries. *Future estimation of food consumption in Japan (2019)*. https://www.maff.go.jp/primaff/seika/attach/pdf/190830_1.pdf (2019).
2. National Institute of Advanced Industrial Science and Technology (AIST), Safety Science Research Division Society, and LCA Research Group. *LCI Database IDEA version 3.1.0.* (2021).
3. Nansai, K., Fry, J., Malik, A., Takayanagi, W. & Kondo, N. Carbon footprint of Japanese health care services from 2011 to 2015. *Resour. Conserv. Recycl.* **152**, 104525 (2020).
4. Ministry of Agriculture Forestry and Fisheries of Japan. Food Loss Statistics Survey (Household Survey and Food Service Industry Survey).

https://www.maff.go.jp/j/tokei/kouhyou/syokuhin_loss/ (2016).

5. MEXT. *STANDARD TABLES OF FOOD COMPOSITION IN JAPAN - 2015 - (Seventh Revised Version)*. https://www.mext.go.jp/en/policy/science_technology/policy/title01/detail01/1374030.htm (2015).

6. Ministry of Internal Affairs and Communications. Family Income and Expenditure Survey. https://www.stat.go.jp/english/data/kakei/index.html (2023).

7. Ministry of Agriculture Forestry and Fisheries of Japan. *The Situation Concerning Meat and Poultry Eggs: Distribution of beef and pork (Shokuniku Keiran wo Meguru Josei; Gyuniku Butaniku no Ryutsu ni Tsuite) (in Japanese)*. https://www.maff.go.jp/j/chikusan/shokuniku/lin/ (2024).
