# Supplementary material for: Curbing household food waste and associated climate change impacts in an ageing society
Source: Nat Commun. 2024 Oct 21;15:8806. doi: 10.1038/s41467-024-51553-w (PMC11494014; doi:10.1038/s41467-024-51553-w)
Supplement: Supplementary file 3 — Description of Additional Supplementary Files [file 41467_2024_51553_MOESM3_ESM.pdf]

## Description of Additional Supplementary Files

File Name: Supplementary Data 1

Description: Table of the sector concordance among statistics utilized in this study

File Name: Supplementary Data 2

Description: Detailed sensitivity results of FW and FWGHG with respect to the FW ratio (a) and edible ratio (b)
