# Supplementary material for: Curbing household food waste and associated climate change impacts in an ageing society
Source: Nat Commun. 2024 Oct 21;15:8806. doi: 10.1038/s41467-024-51553-w (PMC11494014; doi:10.1038/s41467-024-51553-w)
Supplement: Supplementary file 6 — Reporting Summary [file 41467_2024_51553_MOESM6_ESM.pdf]

nature portfolio

Corresponding author(s): Yosuke Shigetomi
Last updated by author(s): Jul 8, 2024

Reporting Summary

Nature Portfolio wishes to improve the reproducibility of the work that we publish. This form provides structure for consistency and transparency in reporting. For further information on Nature Portfolio policies, see our Editorial Policies and the Editorial Policy Checklist.

Statistics

For all statistical analyses, confirm that the following items are present in the figure legend, table legend, main text, or Methods section.

n/a	Confirmed	
☐	☒	The exact sample size (*n*) for each experimental group/condition, given as a discrete number and unit of measurement
☒	☐	A statement on whether measurements were taken from distinct samples or whether the same sample was measured repeatedly
☒	☐	The statistical test(s) used AND whether they are one- or two-sided<br>*Only common tests should be described solely by name; describe more complex techniques in the Methods section.*
☒	☐	A description of all covariates tested
☐	☒	A description of any assumptions or corrections, such as tests of normality and adjustment for multiple comparisons
☐	☒	A full description of the statistical parameters including central tendency (e.g. means) or other basic estimates (e.g. regression coefficient) AND variation (e.g. standard deviation) or associated estimates of uncertainty (e.g. confidence intervals)
☒	☐	For null hypothesis testing, the test statistic (e.g. *F*, *t*, *r*) with confidence intervals, effect sizes, degrees of freedom and *P* value noted<br>*Give P values as exact values whenever suitable.*
☒	☐	For Bayesian analysis, information on the choice of priors and Markov chain Monte Carlo settings
☒	☐	For hierarchical and complex designs, identification of the appropriate level for tests and full reporting of outcomes
☒	☐	Estimates of effect sizes (e.g. Cohen's *d*, Pearson's *r*), indicating how they were calculated

Our web collection on statistics for biologists contains articles on many of the points above.

Software and code

Policy information about availability of computer code

Data collection	All data was downloaded manually.
Data analysis	Matlab 2024a and Microsoft Excel for Microsoft 365 were used.

For manuscripts utilizing custom algorithms or software that are central to the research but not yet described in published literature, software must be made available to editors and reviewers. We strongly encourage code deposition in a community repository (e.g. GitHub). See the Nature Portfolio guidelines for submitting code & software for further information.

Data

Policy information about availability of data

All manuscripts must include a data availability statement. This statement should provide the following information, where applicable:

- Accession codes, unique identifiers, or web links for publicly available datasets
- A description of any restrictions on data availability
- For clinical datasets or third party data, please ensure that the statement adheres to our policy

All of the data for the analysis were described in the Supplementary File.

Research involving human participants, their data, or biological material

Policy information about studies with human participants or human data. See also policy information about sex, gender (identity/presentation), and sexual orientation and race, ethnicity and racism.

Reporting on sex and gender	N.A.
Reporting on race, ethnicity, or other socially relevant groupings	N.A.
Population characteristics	N.A.
Recruitment	N.A.
Ethics oversight	N.A.

Note that full information on the approval of the study protocol must also be provided in the manuscript.

Field-specific reporting

Please select the one below that is the best fit for your research. If you are not sure, read the appropriate sections before making your selection.

☐ Life sciences ☐ Behavioural & social sciences ☒ Ecological, evolutionary & environmental sciences

For a reference copy of the document with all sections, see nature.com/documents/nr-reporting-summary-flat.pdf

Ecological, evolutionary & environmental sciences study design

All studies must disclose on these points even when the disclosure is negative.

Study description	This study presents the quantitative structure of household food waste and the associated life cycle greenhouse gas emissions across households according to the age of the household head in Japan.
Research sample	The average household consumption expenditure and those for the age brackets were retrieved from the Family Income and Expenditure Survey (FIES) and the National Survey of Family Income and Expenditure (NSFIE), Japan. The average food prices by food item were retrieved from the Retail Price Survey (RPS). The average food waste ratio from households by food item were retrieved from the Food Loss Statistics Survey (FLSS). The edible ratio of food in the retail stage were calculated from the Standard Tables of Food Composition in Japan (STFC). The demographic data (the number of households and the average number of household sizes for the age brackets) were retrieved from the database on the National Institute of Population and Social Security Research (IPSS) and NSFIE. The life cycle climate impacts (GHG emissions) for the food wastes were retrieved or calculated from the Inventory Database for Environmental Analysis (IDEA) and the Embodied Energy and Emission Intensity Data for Japan Using Input-Output Tables (3EID). The relevant sample numbers on these data are described in the manuscript.
Sampling strategy	This study did not perform sampling-size calculation because all of the research samples were based on the public statistics.
Data collection	All of the research samples of this study can be collected from the websites of the corresponding statistics as the Excel or CSV format.
Timing and spatial scale	The centrally analyzed year is 2015 and the spatial scale is a whole of Japan, both regarding the currently latest and available dataset on the public statistics and database. Specifically, the FIES, RPS, STFC, IDEA, 3EID, IPSS cover the 2015 data. The NSFIE and FLSS cover the 2014 data, which is closest to the 2015. The projection were also made from 2016 to 2040 based on the future numbers of households for the age brackets published by the IPSS.
Data exclusions	No data were excluded.
Reproducibility	All methods are described in the main manuscript and supplementary information, ensuring reproducibility.
Randomization	This is not relevant because all the research samples were based on the public statistics.
Blinding	Because the inventory data on the IDEA is prohibited to disseminate, the specific results of food waste related GHG emission are blinded.

Did the study involve field work? ☐ Yes ☒ No

Reporting for specific materials, systems and methods

We require information from authors about some types of materials, experimental systems and methods used in many studies. Here, indicate whether each material, system or method listed is relevant to your study. If you are not sure if a list item applies to your research, read the appropriate section before selecting a response.

Materials & experimental systems

n/a	Involved in the study
☒	☐ Antibodies
☒	☐ Eukaryotic cell lines
☒	☐ Palaeontology and archaeology
☒	☐ Animals and other organisms
☒	☐ Clinical data
☒	☐ Dual use research of concern
☒	☐ Plants

Methods

n/a	Involved in the study
☒	☐ ChIP-seq
☒	☐ Flow cytometry
☒	☐ MRI-based neuroimaging

Plants

Seed stocks	N.A.
Novel plant genotypes	N.A.
Authentication	N.A.
